# Ethnobotanical Survey of *Dracaena cinnabari* and Investigation of the Pharmacognostical Properties, Antifungal and Antioxidant Activity of Its Resin

**DOI:** 10.3390/plants7040091

**Published:** 2018-10-26

**Authors:** Mohamed Al-Fatimi

**Affiliations:** Department of Pharmacognosy, Faculty of Pharmacy, Aden University, P.O. Box 5411, Maalla, Aden, Yemen; alfatimi@web.de

**Keywords:** *Dracaena cinnabari*, ethnobotany, resin, organoleptic, extractive value, antifungal, antioxidant, Soqotra

## Abstract

*Dracaena cinnabari* Balf. f. (Dracaenaceae) is an important plant endemic to Soqotra Island, Yemen. Dragon’s blood (*Dam Alakhwin*) is the resin that exudes from the plant stem. The ethnobotanical survey was carried out by semi-structured questionnaires and open interviews to document the ethnobotanical data of the plant. According to the collected ethnobotanical data, the resin of *D. cinnabari* is widely used in the traditional folk medicine in Soqotra for treatment of dermal, dental, eye and gastrointestinal diseases in humans. The resin samples found on the local Yemeni markets were partly or totally substituted by different adulterants. Organoleptic properties, solubility and extractive value were demonstrated as preliminary methods to identify the authentic pure Soqotri resin as well as the adulterants. In addition, the resin extracts and its solution in methanol were investigated for their *in vitro* antifungal activities against six human pathogenic fungal strains by the agar diffusion method, for antioxidant activities using the DPPH assay and for cytotoxic activity using the neutral red uptake assay. The crude authentic resin dissolves completely in methanol. In comparison with different resin extracts, the methanolic solution of the whole resin showed the strongest biological activities. It showed strong antifungal activity, especially against *Microsporum gypseum* and *Trichophyton mentagrophytes* besides antioxidant activities and toxicity against FL-cells. These findings confirm and explain the traditional uses of the resin for the treatment of skin diseases and mouth fungal infections.

## 1. Introduction

**Figure 1:**
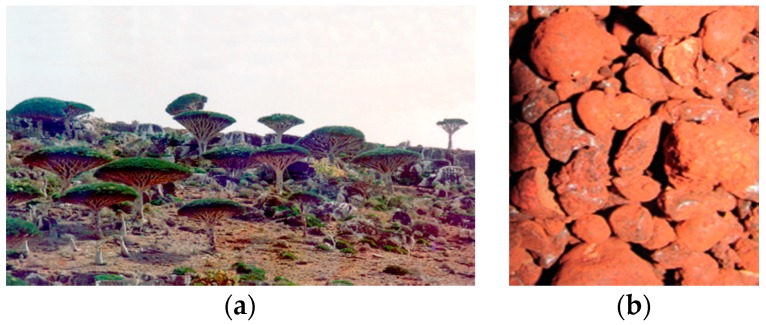
The ecological habitat (**a**) and the pure raw resin (**b**) of *D. cinnabari* in Soqotra Island (Author’s pictures 1990).

Dragon’s blood is the English common name which has been applied to the red coloured resins that are obtained from six different plant genera, *Dracaena*, *Daemonorops*, *Calamus*, *Pterocarpus*, *Eucalyptus* and *Croton* [1,2,3]. Besides *Dracaena cinnabari*, the genus *Dracaena* is represented by some other species, e.g., *D. ombet* and *D. serrulata* that are distributed in tropical Africa and southwest Arabia and *D. draco*, *D. tamaranae* from the Canary Islands and Morocco [4]. *D. cinnabari* Balf. f. (Dracaenaceae) is endemic to Soqotra Island, Yemen (Figure 1), in which more than 308 plant species have been considered to be endemic [4,5]. The resin of *D. cinnabari* from Soqotra is well known as a traditional remedy with a long history [4]; it has been traded as medicine and as a colourant agent for use in works of art for centuries, e.g., it has been discovered in some art works in a museum in Germany [3]. The original source of dragon’s blood resin is believed to be *D. cinnabari* from Soqotra [1] (Figure 1) but numerous different resins produced from different plant species are available on the international markets under the name of Dragon’s blood [3]. Therefore, some methods have been studied to differentiate these species red resins, e.g., solubility, gas chromatography coupled to mass spectrometry [3], Raman spectroscopy [1,2] and UV spectroscopy. Flavylium chromophores (7,4′-dihydroxyflavylium) [6], have been identified to be responsible for the red colour of *D. cinnabari* resin, while other flavylium derivatives are responsible for the red color of the other species resins that are also called Dragon’s blood [6]. The Soqotri resin is the most famous crude drug used in the traditional medicine in Soqotra Island [4,5,7]. Moreover, it is imported from Soqotra in order to be used in other traditional medicine systems all over the world, especially for the treatment of skin diseases [2,4], beside other Dragon’s blood resins that have been reported in different traditional medicine systems in the world [4,8]. 

Some polyphenolic derivatives such as chalcones, homoisoflavans, flavones, biflavonoids (Cinnabarone), triflavonoids (Damalachawin) and metacyclophanes (dracophane) have been identified in the resin of *D. cinnabari* besides many sterols such as campesterol, stigmasterol, sitosterol and stigmastanol [6,9,10,11,12,13,14]. Concerning its biological activities, antioxidant and cytotoxic properties have been reported [7,9,15]. Our previous study [7] reported the marked cytotoxic activity of a methanolic solution of the resin of *D. cinnabari* on the human ECV-304 cell line (human bladder carcinoma cell line with endothelial properties) [7].

In the course of our biological and phytochemical studies of medicinal plants from Yemen [5,7,16], we investigated the resin of *D. cinnabari.* Different ethnobotanical data about *D. cinnabari* have been reported by Al-Fatimi [5] and by Miller [4]. Those are compared with our ethnobotanical survey in the present study. The aim of this study was to document the ethnobotanical data and the traditional medicinal and non-medicinal uses of the plant and its resin. The pharmacognostical and organoleptic properties of the *D. cinnabari* resin were determined for the first time in this study. These findings can be used for the identification and quality control of the resin in case of adulterations. In addition, the *in vitro* antifungal activity of the resin against some human pathogenic fungal strains and its antioxidant activity have been investigated to confirm the traditional medicinal uses.

## 2. Results and Discussion

### 2.1. Ethnobotany of Dracaena cinnabari

#### 2.1.1. Botanical Data

*Dracaena cinnabari* Balf. f. (Dracaenaceae) is the botanical name of an endemic wild tree, growing on Soqotra Island (Figure 1). Dragon’s blood is the English common name for both tree and resin. The Arabic name “*Dam Alakhwin*” means “Brother’s blood” and is also used for both tree and its resin (Figure 1, Table 1). In contrast, there are two different Soqotri names for the tree and its resin. The local Soqotri name of the endemic plant (tree) is *“Ahrrebo*”, while the Soqotri name of the authentic pure superior resin is *“Emzoloh*” (Figure 1, Table 1). All informants (100%) reported the two Soqotri local names of the plant and the resin. Only 40% held knowledge about the Arabic name *“Dam Alakhwin*”. Nearly all informants (95%) reported that the authentic resin with the best quality “*Emzoloh*” exudes from the stem bark of the female tree of *D. cinnabari*, while 5% reported both male and female trees as source for the resin.

#### 2.1.2. Ethnobotanical Uses

The different ethnobotanical uses of *D. cinnabari* resin in the study area can be classified into two main groups: medicinal and non-medicinal uses (Table 2). All informants reported the two main groups of the plant resin uses.

- Non-medicinal uses: The non-medicinal uses of the resin were divided into two categories—cosmetic and dye uses—according to ethnobotanical data. 95% of the informants reported the use of resin by women as lipstick and nail varnish and as smoothing and softening agent for women faces. All informants reported the use of the red resin as colourant agent for bottles and pottery vessels. No informant reported the use of the plant as food source for humans but 10% of the informants reported its fruit as food for livestock (Table 2).
- Medicinal uses: The field survey on *D. cinnabari* was conducted to provide information about established applications of the plant in the traditional medicine. 85% of the informants rely on traditional uses of the endemic medicinal plants of the island. All the local informants (100%) reported medicinal uses of the plant resin in Soqotra. The survey indicated wide uses of the resin against different ailments. Twenty diseases were reported to be treated using *D. cinnabari*. These ailments were classified into five categories as summarized in Table 2. Dermal diseases and dental ailments are most frequently treated with the resin (100% responses, for each), followed by eye diseases (98%), internal and external bleeding (95%) and gastrointestinal diseases (94%) (Table 2). Treatment of wounds and dental diseases are reported in the present study for the first time (Table 2). The resin of the endemic *D. cinnabari* is used extensively in Soqotra and also in inland Yemen as powerful medicinal herb [4,5]. This survey confirmed some medicinal uses that have been already reported, such as treatment of skin diseases, stomach pain, bleeding and inflamed eye [4,5,7]. Among the informants, knowledge about toxic effects of the resin is very limited. 80% of the informants reported an abortive effect in pregnant women.

#### 2.1.3. Used Plant Part

All informants (100%) reported the resin as dominant part used of the plant; only 5% of them reported the leaves as additional part used (Table 2).

#### 2.1.4. Modes of Preparation

The preparations (traditional forms) are made from dried (50% of preparation modes) or fresh resin (50%): (A) Dried resin can be used as powder (40% of preparation modes) or as suspension (33%). The dried resin is crushed to powder or suspended in little water. (B) Fresh resin is crushed with little water to prepare a paste (20%). For internal use, fresh resin is chewed or its powder is taken (Table 2). Powdered resin may be mixed with curcuma or antimony for external use while it is suspended in milk for internal use (Table 2). The usual preparation mode is powdered dried resin.

#### 2.1.5. Quantitative Analysis of Ethnobotanical Data

Ethnobotanical knowledge about *D. cinnabari* is very similar among the local people in Soqotra Island, therefore similar reports were collected from all informants. The Use Value (UV) demonstrates the relative importance of *D. cinnabari* among indigenous people in Soqotra Island. According to the quantitative analysis, the use value index of *D. cinnabari* is high (1.95) (Table 3).

The Fidelity Level (FL) is calculated to indicate the efficiency of *D. cinnabari* to cure a given ailment; it assesses the informants’ consensus about the therapeutic value of a plant species [17]. According to the calculated FL levels that range between 55–100% (Table 3), *D. cinnabari* is identified as an important medicinal plant species in the traditional medicine in Soqotra to treat various ailments. The endemic plant showed highest FL levels for the treatment of skin and head sores (FL = 100%), followed by conjunctivitis (95%), tooth decay (94%), bleeding (postpartum haemorrhage) 85% and stomach pain (55%) (Table 3). These data may serve as a guide for further biological investigations [18] in order to isolate bioactive compounds.

### 2.2. Adulteration Types of D. cinnabari Resin

#### 2.2.1. Soqotri Standard Resin

The Soqotri resin (Dragon’s blood = *Dam Alakhwin*) is the pure red blood resin with high quality that is known on the Island under the Soqotri name “*Emzoloh*”. It is collected from the incision of the young stem bark of the female tree; we can describe this standard pure resin as an authentic superior Soqotri resin. The organoleptic and some physical properties of the resin were determined in this study. Like other important crude drugs, the Soqotri authentic resin may be adulterated [19]. Samples were found on the local markets in Soqotra and in inland Yemen that were adulterated by different substitutes (adulterants). Those can be classified into two main adulteration types: adulteration by partial substitution and/or by total substitution.

#### 2.2.2. Adulteration by Partial Substitution

Three partial adulterations types were found in this survey. We can describe these samples according to types of adulteration for crude drugs described in pharmacopoeias [19].

- A sample was a dark resin found on the markets in Soqotra Island and Aden with low quality. This resin type was mixed with bark pieces and sold as second value resin; in case of very low production of the pure resin (Table 4). We can describe this resin product as inferior resin.
- If the resin is mixed intentionally with bark pieces to sell as pure resin “*Emzoloh*”, in this case it is described as a sophistication adulteration type (Table 4).
- Sometimes the resin is adulterated by accident or careless addition of small pieces of stem barks; this product can be described as an admixture adulteration.

#### 2.2.3. Adulteration by Total Substitution

On the other hand, two samples were found in the markets of Aden and Sana’a city which both were totally substituted by red adulterants, which were non-resinous products; each was available in the markets under the name “*Dam Alakhwin*” (Table 4).

The red coloured powder used as adulterant may have two natural origins: a plant root from Soqotra and a red coral from Indian Ocean (Table 4). Both adulterants were found on the markets of crude drugs in the inland but not on Soqotra Island. Both were sold as red power under the name “*Dam Alakhwin*” (Dragon’s blood)

- *Euclea divinorum* Hiern (Ebenaceae) grows on Soqotra Island. The plant is locally named *“Kala*”; its dried roots are used traditionally in Soqotra as toothbrush or as red powder for cleaning teeth and as mouthwash antiseptic agent [7]. However, the plant root and/or its red powder is imported from the Island to the inland of Yemen including Aden City markets, where the dried root pieces and the red powder were available under the name of “*Dam Alakhwin*” (Table 4).
- A red powder of a red coral from Indian Ocean was found on the market in Sana’a City, called “*Dam Alakhwin*” and used for tooth cleaning (Table 4).

The total substitution by adulterants like these is known as substitution adulteration in the pharmacopoeias [20] (Table 4).

- The authentic Soqotri resin may be substituted totally by other red resins obtained from other plant species that each have the same name “Dragon’s blood” and are found on the international markets.

Therefore, further morphological, organoleptic, chemical, physio-chemical and spectroscopical methods must be studied to identify and authenticate the Soqotri authentic pure resin and the adulterants. The pharmacopoeias contain a paragraph for important official crude drugs that involve the usually known adulteration types that they may be found on the international markets [19]. The documentation of common adulteration types can help to identify the authentic pure Soqotri resin either for the medicinal use or for research purposes. 

### 2.3. Pharmacognostical Study

#### 2.3.1. Organoleptic Properties of the Resin

The Soqotri resin is an exudate product obtained from the stem of female *D. cinnabari* tree. The suitable time of collection is from February to March. This valuable pure resin with high quality can be described as superior authentic Soqotri resin. The dried resin is a hard, transparent brittle crystalizable exudate in lump or tear like form. Its colour is glossy deep red to purple (Figure 1). There is a second resin product with low quality that has a lump form containing bark pieces in the resin. This inferior resin is a dark red and hard amorphous material (Table 4). Dried pure resin is very weakly volatile and without characteristic smell, taste is weakly mucilage. These findings are due to the neutral character of the resin. Its suspension in water shows pH value 5.9. The organoleptic properties are an important primary examination for the quality control of crude drugs [20], therefore these results can be used in the identification and authentication of the Soqotri original superior resin.

#### 2.3.2. Extractive Value of the Resin

The determination of the alcohol-soluble and water soluble extractive values is used for quality control of crude drugs [19,20]. Five solvents with increasing polarity were used for determination of the extractive values of two resin types of *D. cinnabari* (superior pure resin and inferior resin) (Table 5). Inferior resin, which is mixed with barks showed different extractive values compared to standard results of the pure superior authentic resin (Table 5). Interestingly, methanol and water extractive values showed clear differentiation between pure and inferior resins. The methanol extractive values were 100% and 90% for pure superior resin and inferior resin, respectively. Water showed no extractive result for superior resin but showed a 10% extractive value for inferior resins. Superior pure resin is dissolved completely in methanol while it is not able to be dissolved in water (Table 5).

#### 2.3.3. Solubility of the Resin

The solubility of the two resin types in different solvents is given by descriptive terms according to the parts of solvent required for one part resin [19] (Table 5). The pure authentic resin of *D. cinnabari* is completely soluble in methanol (one part of solvent required for one part resin), this equals to the descriptive term “easily soluble” in methanol but it is “insoluble” in water and n-hexane. In contrast to the pure resin, the inferior resin is not completely soluble in methanol, due to its bark pieces content. According to the required parts of methanol, it is “freely soluble” in methanol while it is “slightly soluble” in water and “very slightly soluble” in n-hexane (Table 5).

The first method to differ and identify these resins by solubility tests using non-polar solvents such as chloroform has been reported 1883 [20]. Historically, various resins known as Dragon’s blood differ widely from one another in their purity and appearance [21]. Generally, resins are known to be dissolved in alcohol but insoluble in water [20], which is consistent with present results of the solubility values of the Soqotri pure resin (Table 5). Therefore, the methanol and water solubility and extractive values can be used as a preliminary test and as important marker to show if the crude drug is a resin, non-resin or adulterated resin (adulterant). If the results are standard, this allows further specific examinations such as spectroscopical methods to identify the resin and its plant species. Moreover, both solubility and extractive values can be employed also for (a) the evaluation the purity of the authentic resin of *D. cinnabari* and, for (b) identification of the adulteration of the pure resin.

### 2.4. Antifungal Activity of the Resin

The FL value of *D. cinnabari* resin to treat skin diseases was the highest value in the survey (Table 3). Therefore, the antifungal activities of the pure resin and its extracts have been studied against six human pathogenic fungi for the first time (Table 6). This study analyzed the antifungal activity of *D. cinnabari* resin by measuring the growth inhibition zone in a paper disc diffusion assay (Table 6). The best antifungal activity was demonstrated by the methanolic solution of the crude resin (20–30 mm) against *A. fumigatus*, *M. gypseum* and *T. mentagrophytes* followed by less polar dichloromethane and ethyl acetate extracts (18 to 20 mm), compared with the antifungal reference nystatin. In contrast, the residue of the resin dissolved in methanol showed weak activity (Table 6). The methanolic solution of the whole resin was evaluated for the MIC. The resin solution showed a MIC at 500 μg/mL with inhibition zones 8 mm and 10 mm towards *M. gypseum* and *T. mentagrophytes* respectively.

Compared to antibacterial activities determined previously [22,23,24], the human pathogenic fungi tested appeared to be more susceptible to the resin extracts than the bacterial stains. Previous studies have reported the antifungal activities of *D. cinnabari* resin against three other species *Candida albicans*, *Aspergillus flavus*, and *A. niger* [22,23,24]. Polyphenols including flavonoids, are the main constituents of the *D. cinnabari* resin [9,10,14] as well as resins obtained from other species of *Dracaena* genus such as *Dracaena cochinchinensis* [8,25], *Dracaena draco* and *Dracaena tamaranae* [26]. Phenolic compounds are known to exhibit antimicrobial activities [20]. Red resin of *Dracaena cochinchinensis* from China has been reported to have antifungal activity [8]. Some other red resins also showed specific antifungal activities such as the resin obtained from *Croton urucurana*, which is called as Dragon’s blood, too [27]. These findings suggest that the Soqotri resin has specific stronger antifungal but lesser antibacterial activity.

### 2.5. Antioxidant Activity

The results of antioxidant assay compared with the effect of ascorbic acid as positive control are shown in Table 7. The methanolic solution of the whole pure resin showed strong antioxidant activity (IC_50_ = 25.5 µg/mL), while the ethyl acetate extract showed considerable antioxidant activity (IC_50_ = 40.5 µg/mL) and the residual methanolic fraction showed weak antioxidant activities (IC_50_ = 153.5 µg/mL), compared to ascorbic acid (15.5 µg/mL) (Table 7). These results indicated that phenolic compounds with low polarity in the ethyl acetate extract are responsible for the main antioxidant activity of the resin, which can be potentiated synergistically by resin compounds contained in the methanolic solution. Polyphenols are known as antioxidant natural products and have also been reported as constituents of *D. cinnabari* resin [9,10,14] and identified as chemotaxonomic marker for species of *Dracaena* genus [8,25,26]. Nevertheless, the antioxidant activity of the methanolic solution of the whole resin was reported in the present study for the first time. Interestingly, the antioxidant activity of the solution of the whole resin dissolved in methanol was stronger than all other polar and non-polar extracts that are reported in this study.

Soqotri resin is a mixture of several phenolic derivatives with high content of flavonoids derivatives [9,10,11,14] that are known as antioxidant compounds [20]. Homoisoflavonoids and tow isolated compounds from the Soqotri resin have been reported for their antioxidant activities, dracophane [14] and a homoisoflavonoid, 7,8-methylenedioxy-3(4-hydroxybenzyl) chromane [15]. 

The strong antifungal and antioxidant activities of the whole raw resin (as solution in methanol) confirm the traditional preparation using the whole crude resin as powder in Soqotra.

### 2.6. Cytotoxic Activity

The results of the cytotoxic activity against the FL-cells, a human amniotic epithel cell line, are shown in Table 7. The resin solution showed low cytotoxicity (IC_50_ = 300.0 µg/mL) against FL-cells measured by the neutral red uptake assay. Extracts showed less cytotoxic activity than the whole resin (IC_50_ > 500.0 µg/mL). These findings confirm the safe uses of the Soqotri resin traditional medicine. The informants in the ethnobotanical survey reported no toxic effects concerning the oral use except by pregnant women where it may cause abortion [5]. 

## 3. Conclusions

*D. cinnabari* is a famous wild endemic plant from Soqotra Island, where all local people use it for various ailments. These results provide pharmacognostical properties to identify and authenticate the Soqotri superior resin and its possible adulterants. Moreover, the ethnobotanical data of the resin led us to investigate its antioxidant and antifungal activities. The solution of Soqotri pure resin in an alcoholic solvent showed the strongest antifungal and antioxidant activities compared to the separated extracts/fractions of this resin. Thus, we suggest that the specific strong biological activities of the resin depend on the synergistic effect of all resin components when it is used completely as methanolic solution. These findings confirm the traditional preparation mode that uses whole crude pure resin as powder and confirm the traditional use of the resin for treatment of skin diseases due to infection with dermatophytes in the tropical island Soqotra.

## 4. Materials and Methods

### 4.1. Ethnobotanical Survey

The interview was conducted during February–March 1990 in ten different localities in Soqotra Island: Hadibu, Momi, Qalansieh, Haggeher, Noged, Mori, Qubah, Qadheb, Dihamidh and Serhin. 100 informants were interviewed (male 90; female 10). Most informants (80%) were within the age group of 35–60 years old who worked in the village. 20% of the informants were students in primary schools between 12–16 years. All informants were indigenous people; they spoke the local Soqotri language and Arabic. Informants had to identify the plant in the nature or the specimen carried during the survey.

### 4.2. Plant Material

*Dracaena cinnabari* plant parts were collected in their natural habitat. The plant was identified at Pharmacognosy Department, Aden University, Yemen. A voucher specimen (No. MF-SOC 001) has been deposited at the Herbarium of the Pharmacognosy Department, Pharmacy Faculty, Aden University, and at the personal Herbarium of the first author.

### 4.3. Data Analysis

Data generated from the field survey on the use of *D. cinnabari* was subjected to descriptive statistics using percentages and frequencies. To quantify the data generated from this survey, the following indices were used: use value index and fidelity level values.

#### 4.3.1. Use Value (UV)

The use value [28,29] is a quantitative method that provides the relative importance of *D. cinnabari* by the local people in Soqotra Island. It was calculated according to the following formula:

UV = ∑U/N  0 < UV < 1

where UV refers to the use value of a species; U to the number of citations per species (*D. cinnabari*) given by informants; and N to the total number of informants.

#### 4.3.2. Fidelity Level (FL)

Fidelity Level (FL) was calculated to determine the percentage of informants that reported the uses of *D. cinnabari* as a remedy for the same major ailment using the formula [17]:

FL% = Np/n × 100

where Np is the number of informants who independently indicated the use of a species for the same major ailment and n the total number of informants who mentioned the plant species for any major ailment [18].

### 4.4. Samples of Standard Rresin and Adulteration Types of Resin

Two types of resins were collected from the endemic wild plant *Dracaena cinnabari* on slopes of Haggeher at Soqotra Island, Yemen, in February 1990 (Figure 1). The superior fresh resin was collected from the young stems of the female tree, and stored as standard authentic resin. The alternative and inferior resin was found on the market in Soqotra Island, it was collected from old stems and mixed with small bark pieces.

The reference sample of pure Soqotri resin *“Emzoloh*” of wild *D. cinnabari* was collected from Soqotra Island. The market samples under the name “*Dam Alakhwin*” = Dragon’s blood were purchased at local markets and suppliers in Soqotra Island, Aden city and Sana’a city. Resin samples were collected from markets in Soqotra Island, Aden and Sana’a. All market samples that were sold under the name of “*Dam Alakhwin*” were compared with authentic standard resin from Soqotra. The taxonomical and organoleptic methods [20] were used for identification of the pure resin or the adulterants.

### 4.5. Extraction of Resin for Biological Tests

The dried pure resin was pulverized in a grinder.

1 g pulverized resin was dissolved completely in 100 mL methanol. This methanol solution (MS) was kept as raw resin solution.

30 g pulverized resin was extracted with a mixture of 10% H_2_O/methanol. This mixture was partitioned against n-hexane (H), dichloromethane (D) and ethyl acetate (E), respectively. Extracts were filtrated and concentrated under reduced pressure at 40 °C. The resin residue remained after extraction was evaporated and dried. The residue was dissolved in methanol (MF) to use for biological evaluation.

Solution and extracts were stored in an exsiccator until use for antifungal, antioxidant and cytotoxicity tests.

### 4.6. Solubility Values

The solubility of the resin in different polar and non-polar solvents was determined according to the solubility test in the pharmacopeia [19].

### 4.7. Extractive Values

n-Hexane, dichloromethane, ethyl acetate, methanol and water-soluble extractive values were determined to find out the amount of polar and non-polar soluble compounds, using two types of the resin that were collected [20].

### 4.8. Antifungal Test (Agar Diffusion Test)

Antifungal activities of the extracts (2 mg dried extract per disc) were determined applying the agar diffusion assay [30]. Nystatin was used as positive control. Inhibition zone diameters include diameter of the disc (6 mm). The test organisms: *Candida krusei* (ATCC 90878), *Absidia corymbifera* (100798), *Aspergillus fumigatus* (13550/99), *Trichophyton mentagrophytes* (05/2004) *Microsporum gypseum* and *Mucor* sp.

### 4.9. Determination of Antioxidant Activity

Qualitative and quantitative estimation of radical scavenging effect were carried out by 1,1-diphenyl-2-picryl-hydrazyl (DPPH) assay. Quantitative estimation was carried out according to the method of Brand et al. [16,31]. Ascorbic acid was used as positive control.

The reaction mixture contained 500 μL of test extract and 125 μL of DPPH in ethanol. Different concentrations of test samples (10, 50, 100, 500 and 1000 g/mL) were prepared while the concentration of DPPH was 1 mM in the reaction mixture. These reaction mixtures were taken in Eppendorf tubes and incubated at 37 °C for 30 min, the absorbance was measured at 517 nm. Percent radical scavenging activity by sample treatment was determined by comparison with ethanol treated control group. Ascorbic acid was used as positive control. The DPPH radical concentration was calculated using the following equation:

Scavenging activity (%) = [(Absorbance control − Absorbance sample)/Absorbance control] × 100

### 4.10. Determination of Cytotoxicity Activity

The cytotoxicity was measured by the neutral red uptake assay [16,32], using FL-cells, a human amniotic epithel cell line. Only living cells are able to manage the active uptake of neutral red. FL-cells were cultivated in a 96-well microtiter plate (105 cell/mL EAGLEMEM, Sifin, Berlin, D, 150 L/well) at 37 °C in a humidified 5% carbon dioxide atmosphere. The EAGLE-MEM was completed by l-glutamin (0.10 g/L), Hepes (2.38 g/L), penicillin G (105 IE/L), streptomycin sulphate (0.10 g/L) and FCS (Gibco, 80.0 mL/L). After 24 h 50 L of the extract solution (extract dissolved in ethanol under stirring in ultrasonic bath for 5 min and then diluted with MEM: test mixture) or medium with equal amounts of ethanol (control) were added. After a further incubation for 72 h cells were washed three times with phosphate buffered saline solution. One hundred microliters of neutral red solution (SERVA, 0.3% in EGLE-MEM) were added per well. The cells were then incubated for 3 h at 37 °C, followed by another three times washing with PBS. One hundred microliters of a solution of acetic acid (1%, v/v) and ethanol (50%, v/v) in distilled water were added. After shaking for 15 min the optical density was measured at 540 nm with an ELISA-Reader HT II (Anthos Labtec Instruments, Salzburg, Austria). The mean of four measurements for each concentration was determine (n = 3).

## Figures and Tables

**Table 1:** Common and local names of *D. cinnabari* tree and its resin.

Name of	Soqotri	Arabic	English
Plant	*Ahrrebo*	*Dam Alakhwin*	Dragon’s blood tree
Resin (fresh)	*Emzoloh*	*Dam Alakhwin*	Dragon’s blood

**Table 2:** Ethnobotanical data of *Dracaena cinnabari* in Soqotra Island.

Ethnobotanical Categories/Uses	PU	AR	Administration Form of Prepared Resin	Ct %	Rf
**1. Medicinal uses**					
**1.1. Dermal diseases**				100	
(a) Acne, rashes, boils, swelling, inflammation (b) Skin and head sores, burn	R	T	(a) Dried resin crushed to powder or mixed with a little water as suspension (b) Fresh resin mixed with a little water and crushed as paste		[4], [or]
Wounds	L	T	Fresh leaves crushed as paste and smear		[or]
**1.2. Dental diseases**				100	
Tooth decay, tooth cleaning	R	T	Fresh powdered resin rubbed on the teeth		[5], [or]
**1.3. Eye diseases**				98	
Eye diseases, conjunctivitis, weak eyesight, eye inflammation	R	T	Dried resin powder: (a) suspended in water and used as eye drops (b) Mixed with powdered antimony to apply		[4,5], [or]
**1.4. Gastrointestinal tract diseases**				94	
Stomach disease, Stomach pain, general tonic	R	O	Fresh resin chewed/ Powder taken		[4], [or]
**1.5. Bleeding (Haemostatic)**				95	
Postpartum haemorrhage, Internal bleeding	R	O	Powdered dried resin mixed with hot milk or mixed with hot water and drunken		[4], [or]
External bleeding	R	T	Powdered dried resin added to water (as suspension) and smeared on affected area		[4], [or]
**2. Non-medicinal uses**					
**2.1. Cosmetic**				100	
Lips stick	R	T	Fresh resin chewed		[4], [or]
To make skin smooth and soft	R	TT	Paste prepared from the mixture of: Fresh resin + curcuma + water; rubbed on the skin		[4]
To clean face, make it smooth and soft, and to disguise moles and freckles from face	R	T	Paste prepared from the mixture of: Fresh resin + curcuma + water; rubbed on the face		[4,5]
**2.2. Dye**				100	
Leather dying	R	-	Powdered dried resin for leather dying		[4]
Decorate pots, pottery and others			Melting dried resin for drawing or colouring		[4], [or]

AR, administration route; T, Topical; O, Oral; [or], Oral interview 1991 in Soqotra Island; PU, Part used; R, Resin; L, Leaf; Ct, citations percentage; Rf, References.

**Table 3:** Quantitative analysis of ethnobotanical data of *D. cinnabari.*

Index	Quantitative Analysis	Importance of Data Analysis
UV = U/N	1.95	Relative importance among people in Soqotra
U = 195		
N = 100		
FL = [Np/n]100 n = 100	100%	Medicinal use of *D. cinnabari* for dermal diseases (skin and head sores)
	95%	Eye diseases (conjunctivitis)
	94%	Dental diseases
	85%	Bleeding (Haemostatic) for postpartum haemorrhage,
	55%	Gastrointestinal tract diseases

UV, use value; U, number of citations of *D. cinnabari* uses given by informants; N, total number of informants; FL, Fidelity Level; Np, number of informants who indicated the use of *D. cinnabari* for the same major ailment; n, the total number of informants who mentioned *D. cinnabari* for any major ailment.

**Table 4:** The adulteration types o the resin of *D. cinnabari.*

Adulteration Type	Adulterant (Substitute)	Market	Local or International Name of Adulterated Product
Inferiority ^a^	small bark pieces	Aden	*Dam Alakhwin*
Admixture ^b^	small bark pieces, small stones	Aden	*Dam Alakhwin*
Sophistication ^c^	small bark pieces	Aden	*Dam Alakhwin*
Substitution ^d^	red powder of *Euclea divinorum* root	Aden	*Dam Alakhwin*
Substitution ^d^	red powder of Indian coral	Sana’a	*Dam Alakhwin*
Substitution ^d^	other red resins from other genera or species	International (India, Iran)	Dragon’s blood

^a^ resin with low quality is collected as alternative for the pure resin due to environmental and economic reasons; ^b^ adulterant is added to resin by accident or carelessness; ^c^ adulterant is added partially and intentionally for marketing; ^d^ resin is substituted totally by other products for marketing.

**Table 5:** Extractive and solubility values of *Dracaena cinnabari* resin.

Solvents	Extractive Value %		Solubility ^c^	
	Pure Resin (Superior) ^a^	Inferior Resin ^b^	Pure Resin (Superior) ^a^	Inferior Resin ^b^
n-Hexane	0.05	5	insoluble	very slightly soluble
Dichloromethane	5	20	sparingly	soluble
Ethyl acetate	30	42	soluble	soluble
Methanol	100	90	easily soluble	freely soluble
Water	00	10	insoluble	slightly soluble

^a^ Obtained from young stem bark; ^b^ Obtained: old stems mixed with small bark pieces; ^c^ descriptive terms are according to pharmacopoeia (according to parts of solvent required for one part solute).

**Table 6:** Antifungal activity of the extracts of the pure resin of *D. cinnabari* in an agar diffusion test.

Microorganisms	Diameter of İnhibition Zone (mm) ^a^					
	H	D	E	MF	MS	Nys
*Absidia corymbifera*	10	10	10	15	15	25
*Aspergillus fumigatus*	15	10	-	-	20	24
*Candida krusei*	-	-	-	10	10	24
*Microsporum gypseum*	15	18	15	-	30	22
*Mucor* sp.	-	10	10	-	10	21
*Trichophyton mentagrophytes*	15	20	18	10	25	25

^a^ Values are inhibition zone diameter (mm) for 2 mg dried extract per disc; -, no inhibition; H, n-hexane; D, dichloromethane extract; E, ethyl acetate extract; MF, residual fraction dissolved in methanol; MS, methanol solution of whole crude resin; Nys, Nystatin (100 µg/disc).

**Table 7:** Cytotoxic activity against FL cells and free radical scavenging activity in the DPPH assay for the solution and extracts of *D. cinnabari* pure resin.

Extracts	IC_50_ (µg/mL)	
	Free Radical Scavenging Activity	Cytotoxicity against FL-Cells
Ethyl acetate	40.5	553.5
Residual fraction (MF) ^a^	153.5	600.4
Methanolic solution of crude resin (MS)	25.5	300.0
Ascorbic acid ^b^	15.5	-

^a^ Residual fraction dissolved in methanol; ^b^ Positive control.
